# Revealing regional variations in scleral shear modulus in a rabbit eye model using multi-directional ultrasound optical coherence elastography

**DOI:** 10.1038/s41598-024-71343-0

**Published:** 2024-09-09

**Authors:** Lupe Villegas, Fernando Zvietcovich, Susana Marcos, Judith S. Birkenfeld

**Affiliations:** 1 Instituto de Óptica, Consejo Superior de Investigaciones Científicas, Madrid, Spain; 2 Department of Engineering, Pontificia Universidad Católica del Peru, Lima, Peru; 3 The Center for Visual Science, The Institute of Optics, Flaum Eye Institute, University of Rochester, Rochester, NY USA

**Keywords:** Sclera, Biomechanics, Shear modulus, Phase speed, Anisotropy, Optical coherence elastography, Biomedical engineering, Biophotonics, Eye diseases, Imaging and sensing

## Abstract

The mechanical properties of the sclera play a critical role in supporting the ocular structure and maintaining its shape. However, non-invasive measurements to quantify scleral biomechanics remain challenging. Recently introduced multi-directional optical coherence elastography (OCE) combined with an air-coupled ultrasound transducer for excitation of elastic surface waves was used to estimate phase speed and shear modulus in ex vivo rabbit globes (n = 7). The scleral phase speed (12.1 ± 3.2 m/s) was directional-dependent and higher than for corneal tissue (5.9 ± 1.4 m/s). In the tested locations, the sclera proved to be more anisotropic than the cornea by a factor of 11 in the maximum of modified planar anisotropy coefficient. The scleral shear moduli, estimated using a modified Rayleigh-Lamb wave model, showed significantly higher values in the circumferential direction (65.4 ± 31.9 kPa) than in meridional (22.5 ± 7.2 kPa); and in the anterior zone (27.3 ± 9.3 kPa) than in the posterior zone (17.8 ± 7.4 kPa). The multi-directional scanning approach allowed both quantification and radial mapping of estimated parameters within a single measurement. The results indicate that multi-directional OCE provides a valuable non-invasive assessment of scleral tissue properties that may be useful in the development of improved ocular models, the evaluation of potential myopia treatment strategies, and disease characterization and monitoring.

## Introduction

The sclera is the fundamental connective tissue, which provides mechanical support to the internal ocular structures (retina, optic nerve head) and, along with the cornea and lens, plays a central role in maintaining the refractive status of the eye^1–3^. Myopia (nearsightedness) results mainly from excessive ocular axial elongation, however, myopia patients may also exhibit a thinner crystalline lens^4,5^ and morphometry differences in the anterior sclera^6,7^ compared to emmetropic subjects.

Although the signaling cascade of myopia presumably triggered by hyperopic defocus and resulting in eye growth is not clearly understood, it is fairly well established that it induces mechanical remodeling of the sclera. In particular, several authors have reported structural changes in the scleral extracellular matrix caused by an up-regulation of metalloproteinase activity and a decrease in the rate of glycosaminoglycans (GAGs) synthesis^8,9^, which may precede thinning and loss of the collagen fiber bundles^1,10^. These microstructural alterations in the sclera lead to biomechanical changes^11,12^, particularly mechanical weakening of the posterior sclera in myopia ^13–16^. Even the healthy sclera is known to show important regional variations in the distribution of proteoglycans and their specific content of GAGs^17^, as well as in the elastic properties^18,19^, and in the orientation of the collagen fibers^20–23^. Despite previous work on animal models and human sclera, non-contact, non-invasive, and non-destructive measurement of the biomechanical properties of the sclera remains a challenge^3,24^.

To date, several techniques have been used to estimate the scleral biomechanical properties of the intact eye^3^. Recent experimental studies have applied inflation testing^20,22,25,26^ and air-puff induced deformation combined with finite element modeling^27,28^ to obtain the dynamic response of the sclera ex vivo. Other studies have attempted to estimate a single stiffness value in different scleral regions. Bronte et al.^28^ combined Optical Coherence Tomography (OCT) air-puff deformation imaging and finite element modeling to estimate material property parameters in porcine sclera and cornea ex vivo*.* Using a hyper-elastic Yeoh material model, this work reported a corneal stiffness value of 0.7 ± 0.1 MPa, while the scleral stiffness ranged from 1.8 ± 0.3 to 6.0 ± 2.1 MPa, depending on the region. Ramier et al*.*^29^ applied Optical Coherence Elastography (OCE) to measure scleral biomechanics in healthy human subjects. In this technique the sclera was excited by a vibrating contact probe placed onto the ocular surface to produce surface waves, the propagation of which is related to the shear modulus of the tissue. This work showed the first in vivo measurements of the shear modulus in the human eye, including the cornea and the anterior, accessible, sclera. The mechanical anisotropy of scleral tissue was not investigated in that work, perhaps because the experimental configuration with lateral excitation is not optimal to capture anisotropy.

There is considerable evidence that the scleral properties are highly anisotropic due to the fiber distribution, making stiffness measurements in the sclera direction-dependent^18,20,23,30^. Previous studies have demonstrated the preferential orientation of collagen fibrils using wide-angle X-ray scattering^30,31^ and inflation testing^22,32^. Regional stiffness values have been extracted from biaxial testing^21,33^ and uniaxial testing^18^ in animal and human scleral tissue. Characterizing scleral anisotropy is important for predicting its response to different mechanical stimuli, as well as an indicator of change in collagen organization, in connection, for example, with myopia^34,35^. However, it has been challenging to experimentally assess scleral anisotropy of intact tissues (whole globe) noninvasively. Recently, emerging techniques, such as Brillouin microscopy^36,37^. Magnetic resonance Elastography^38^ and Optical Coherence Elastography^39^, have been applied to quantify corneal biomechanics. Dynamic OCE imaging is a versatile technique that can be combined with different stimuli^40^ such as micro-air-puff stimulation^41,42^, piezoelectric excitation^29,43^, acoustic micro-tapping^44–46^, sound excitation^47–49^ and air-coupled ultrasonic (ACUS) excitation^50,51^ to detect mechanical wave propagating in the ocular tissue. In studies on the ex vivo porcine sclera, OCE has been applied on the anterior and posterior scleral surface using low^43^ and high^52^ excitation frequencies, estimating wave speed values around 10 m/s at 0.8 kHz (anterior sclera), and 21.6 ± 2.5 m/s at 16 kHz (posterior sclera). Thus, wave-based OCE has demonstrated its feasibility to account for the shear modulus of tissue and could be a useful tool for non-invasive in situ mapping of scleral stiffness. Additionally, its capability of measuring mechanical properties of the sclera in conditions that maintain its physiological integrity similar to in vivo conditions is fundamental for basic research and facilitates its clinical translation.

In this article, we report, for the first time to our knowledge, quantitative, non-contact measurements of scleral biomechanics and anisotropy on a rabbit eye model, using a multi-directional (8 radial directions) air-coupled ultrasonic OCE (ACUS-OCE) imaging approach. In our experimental implementation, the ultrasonic excitation is focally produced on the ocular tissue and a multi-directional acquisition configuration allowed a radial mapping of the phase speed (which is the wave speed at a specific frequency) of the resulting elastic surface waves, for the first time within a single measurement. In addition, the Young´s modulus was obtained from strip extensiometry for comparison to the shear modulus. Our study included seven enucleated rabbit eyes that were measured at four different scleral locations, and at the corneal apex. Our results revealed a faster and directional-dependent phase speed on the scleral surface in comparison to the cornea, giving insights into the scleral mechanical anisotropy. We also demonstrated that the estimated shear moduli were largest in the anterior scleral zone and smallest in the posterior zone, but no correlation was found between the estimated shear and Young´s modulus in the investigated corneal and scleral locations.

## Methods

Eyes were prepared, measured with multi-directional OCE system, then strips were cut and extensiometry was performed. In the following, all experimental methods are described in detail:

## Sample preparation

**Fig. 1:**
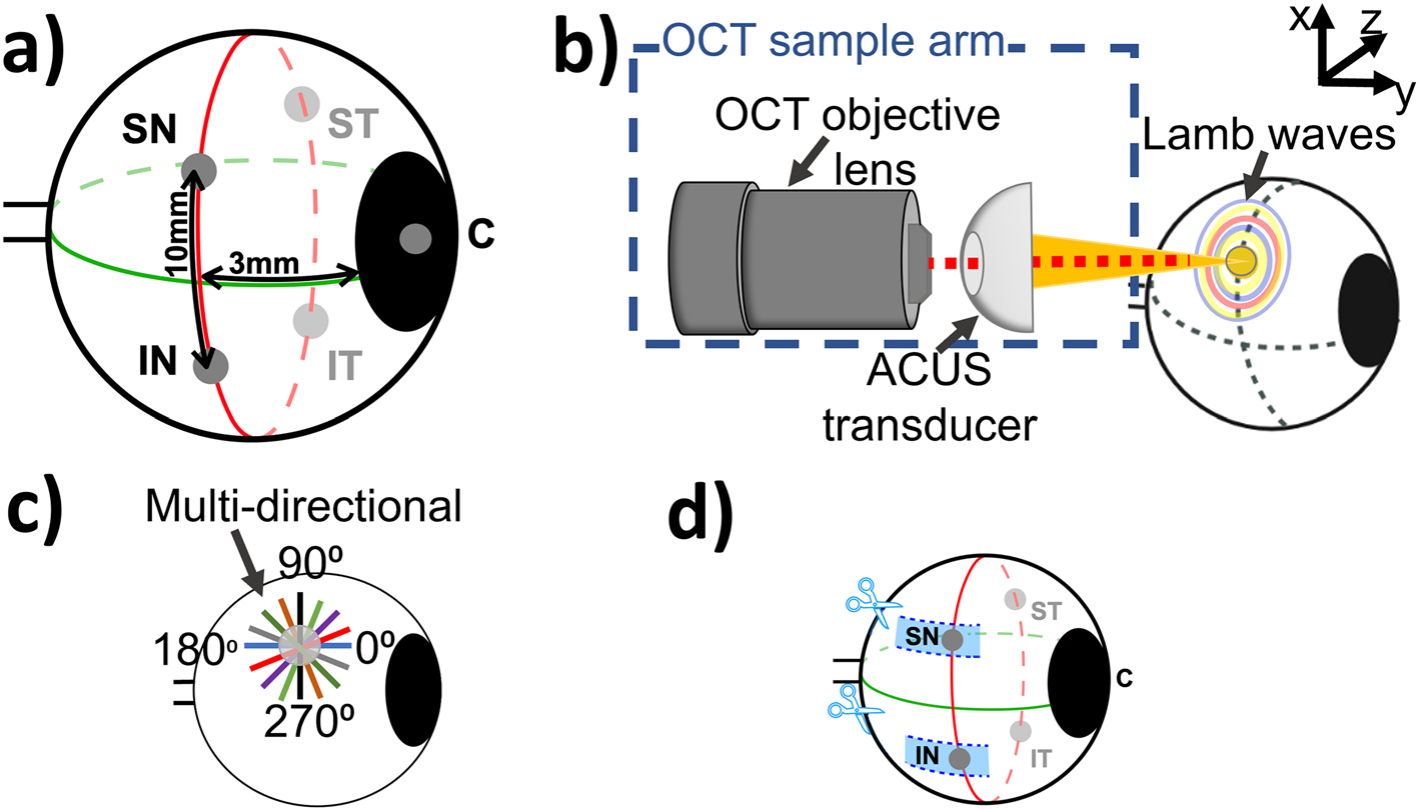
Schematic representation of the measurement method. **(a)** Diagram of ocular globe showing investigated corneal (C) and scleral locations: superior nasal (SN), inferior nasal (IN), superior temporal (ST), and inferior temporal (IT). The equatorial circumferential axis of the eye is shown in red, and the nasal-temporal meridional axis in green. The equatorial circumferential axis divides the eye into anterior and posterior scleral zones. (**b)** Schematic of OCE system implementation. The ACUS transducer was aligned with the OCT sample arm to acquire multiple scans while the transducer induced elastic waves at the described locations. Lamb waves propagate on the tissue surface at the same time as the tissue is scanned by the OCT system. (**c)** The colored lines indicate the 8 acquisition axes (radial directions) of the OCT system in a schematic eye. (**d)** After OCE measurements, sclera strips were cut along meridional direction (dashed blue lines).

Seven freshly enucleated rabbit eyes from adult New Zealand white rabbits (2–3 kg) were obtained (Facultad de Veterinaria, Complutense University of Madrid) and used within 30 h *post-mortem*. The rabbits were euthanized by cervical dislocation in the context of veterinary activities ((CEA)‐UCM‐5414122021‐2021). Before measurements, muscles and conjunctival tissue were removed from the sclera, and five locations (1 corneal, 4 scleral) were identified and marked: central cornea (C), and superior nasal (SN), inferior nasal (IN), superior temporal (ST), and inferior temporal (IT) sclera (Fig. 1a). The scleral locations were defined at 3 mm away from the limbus along the nasal-temporal meridional line towards the optic nerve (green line, Fig. 1a), and 5 mm away along the equatorial circumferential axis (red line, Fig. 1a). The corneal location was defined at the corneal apex. After preparation, the intact eye was placed in a customized holder and aligned with the OCT laser scanning beam. A 25-gauge needle connected to a pressure control system was inserted through the optic nerve head to maintain the eye at a constant intraocular pressure (IOP) of 15 mmHg during the measurements. After the OCE measurements, scleral and corneal strips were extracted from the globes within 24 h and mechanically stretched. All measurements were completed on the same day.

### Multi-directional optical coherence elastography system

OCE measurements were performed using a phase-sensitive swept-source optical coherence tomography system (PhS-SS-OCT), combined with an air-coupled ultrasonic transducer for tissue excitation^51^. The PhS-SS-OCT is a custom-built system reported in previous publications^49,53^ featuring a swept source (SL132120, Thorlabs, Newton, NJ, USA) centered at 1300 nm (VCSEL) with a spectral 3 dB-bandwidth (50 nm) and an axial scan rate of 200 kHz. The OCT axial and transverse resolution in air is 16 μm and 40 µm (at the focal plane), respectively. PhS-OCT mode^49^ was used to obtain the sub-resolution sample displacements over time in response to a stimulation. The system provides multi-directional scanning using high-speed galvanometric scanning mirrors (Saturn 1B, Scanner MAX, Pangolin, USA). We used this functionality to acquire 8 radial directions of 15 mm length (16 semi-axes) equally distributed over 360 degrees (0°–337.5° in steps of 22.5°) (see Fig. 1c).

The acoustic transducer was co-focused and co-axially aligned with the sample arm of the PhS-SS-OCT by means of a 15 mm diameter aperture through which the OCT laser beam could pass. This set-up allowed the acquisition of multiple scans of wave propagation while the transducer induced elastic waves on the ocular tissue (Fig. 1b). The acoustic beam had a lateral spot size of approx. 0.6 mm. The transducer was excited with a 3-cycle 2 kHz train of square pulses modulating at 0.5 MHz signal which was amplified with a radiofrequency amplifier (100A250A, Amplifier Research, USA).

### OCE measurements

For this work, a multi-directional M-B scanning protocol^41^ was introduced during the acoustic stimulation to capture the tissue excitation in 8 radial directions. In our protocol, 450 A-scans were acquired for high temporal resolution with Δt = 5 µs at each spatial point. This M-mode scan was repeated for 100 spatial positions along each B-scan, which consisted of 45,000 A-scans and covered the transverse range of 15 mm with 0.15 mm spatial resolution. The B-scans were acquired in 8 radial directions with a rotation step of 22.5 degrees from the horizontal direction centered at the corneal location or the transversal direction (meridional planes) at the scleral locations, as previously described in Fig. 1c. The total acquisition time was 1.8 s, including the 8 M-B-mode radial scans centered at each location in Fig. 1a. Eyes were located in a custom-made holder and the locations (C, SN, IN, ST or IT) were aligned to the OCT sample arm (Fig. 1b). Samples were excited to generate elastic waves on the sample surface. OCE measurements were performed at the five locations (Fig. 1d) in each eye using the scan pattern. Each eye location was measured three times, with a time separation of 30 s between measurements to avoid wave coupling. The eye was kept hydrated with drops of saline solution between measurements. Phase speed and thickness values were obtained from the images taken by the OCT system for each location (see examples of OCT images in Supplementary Figs. 5–7) and each of the 16 angles. Because data were obtained from both left and right eyes, all results were inverted with respect to the vertical axis to be considered as coming from left eyes.

### Phase speed, tissue thickness, and shear modulus calculation

**Fig. 2:**
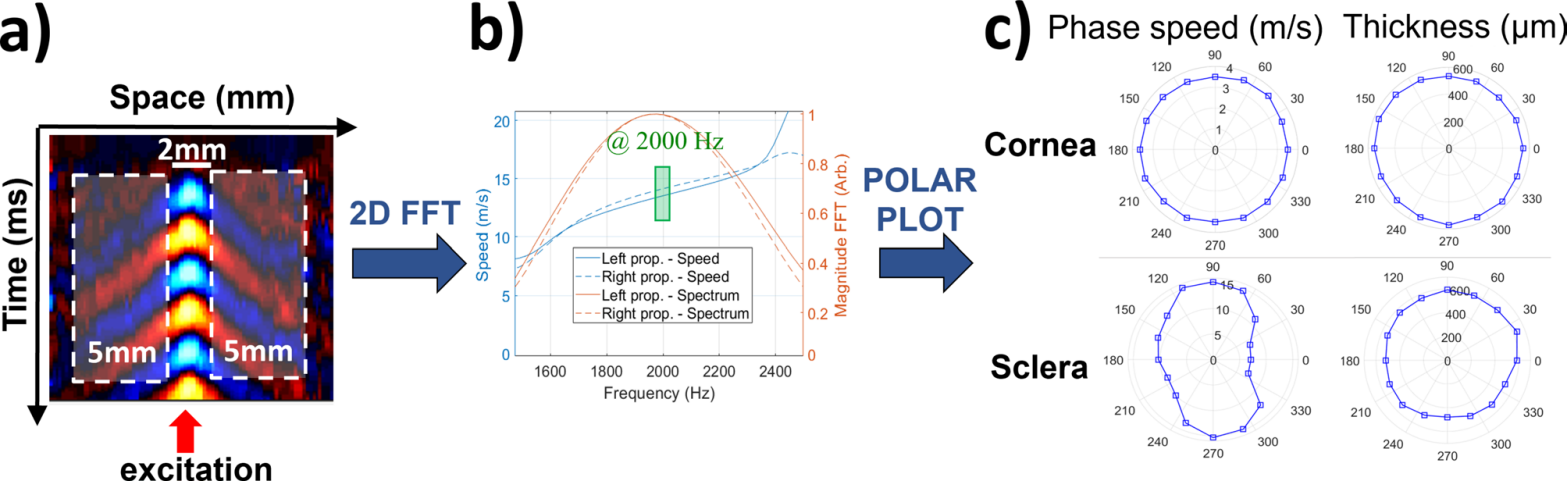
Corneal and scleral phase speed and thickness estimation. **(a)** Example of a spatio-temporal map of wave propagation in the ocular tissue along one direction. The ultrasound excitation point is indicated by the red arrow. The dashed boxes (5 mm size along time) represent the boundaries used to calculate the phase speed. (**b)** The calculated speed from a) (blue), together with the Magnitude FFT (orange) in function of the frequency, allows the phase speed estimation at a frequency of 2 kHz. (**c)** Phase speed and thickness are presented in polar plots and evaluated in the measured 16 semi-axes.

The phase speed of the Lamb wave speed^54^ was quantified at both corneal and scleral locations using the previously reported Fourier estimators^41,51^. In short, the phase speed calculation is performed in the spatio-temporal domain (Fig. 2a) which is converted into the spectral domain by applying the 2D Fourier transform (see Fig. 2b). Subsequently, the transformed signal is evaluated at the excitation frequency of $\omega =2\pi \cdot 2000 \text{Hz}$ to estimate the phase speed per each of the 16 semi-axes. Polar plots were used to present the phase speed (in m/s) as a function of the wave propagation angle measured, and to give a visual assessment of tissue anisotropy (Fig. 2c).

Both corneal and scleral thicknesses were determined from automatic segmentation of B-mode OCT images using an edge detection algorithm^51^ at each tested location (C, SN, IN, ST, IT). The algorithm detected the external and internal surface of the tissue and measured the averaged thickness within a 5 mm lateral window located at each semi-axis. The measured thickness was corrected by the corresponding refractive indices of cornea ${n}_{cornea}\sim 1.376$, and sclera ${n}_{sclera}\sim 1.42$^55,56^. The shear modulus was estimated using the modified Rayleigh-Lamb frequency equation (mRLFE) model^43,54^. This approach has been applied to phase speed data assuming, as first approximation, that corneal and scleral tissue are isotropic, homogenous, and viscoelastic material^51^. Although this model does not fully account for the anisotropy of the data since it will require a priori information of scleral biomechanics, it has been assumed tissue isotropy in a constrained region of interest and propagation direction as a first approximation to quantify direction-dependent elastic moduli for interrogating scleral anisotropy. The shear modulus was calculated from phase speed and thickness data obtained from OCE measurements at each tested location and on each of the 16 semi-axis (8 directions).

The data of scleral phase speed were pooled and grouped in anterior, and posterior zones, and meridional and circumferential orientations. The anterior zone was defined as the semi-axes at angles 0°, 22.5°, and 337.5° (SN/IN locations), and semi-axis at angles 157.5°, 180° and 202.5° (ST/IT locations). The posterior zone was defined as the semi-axes at angles 157.5°,180°,202.5° (SN/IN locations), and 0°, 22.5°and 337.5° (ST/IT locations).

To compare results between meridional and circumferential orientations, the meridional zone was defined with the semi-axes at angles 0°, 22.5°, 157.5°, 180°, 202.5°, and 337.5°, and the circumferential zone at angles 67.5°, 90°, 112.5°, 247.5°, 270°, and 292.5°. The mean phase speed of the cornea was calculated as the mean of the corneal phase speed data at all angles.

### Mechanical anisotropy parameters

Using the calculated phase speed, three anisotropy parameters were assessed, (1) the normalized fractional anisotropy (NFA)^42^, (2) the maximum of modified planar anisotropy coefficient (maxMPAC)^42^, and (3) the major-axis angle (MAA) of the phase speed polar plots. The normalized fractional anisotropy (Supplementary equation (G.1)) is a single estimation of the anisotropy degree using phase speed values at each location. NFA values close to zero show smaller speed differences and low anisotropy, while values close to 1 are associated with greater differences and high anisotropy. The planar anisotropy coefficient (Supplementary equation (G.2)) represents the spatial anisotropy. MPAC was calculated using the phase speed as a measure of the planar-transverse strain ratio at each semi-axis. MPAC values greater than 1 (high anisotropy) correspond to an “earing” behavior (the ripple at the edge of the polar graph that represents the effects of speed in planar anisotropy^57^). Finally, the major-axis angle in the phase speed polar plots in all scleral locations was obtained using principal component analysis. The MAA corresponds to the direction of the first principal axis measured from the corneal limbus to the optic nerve head.

### Uniaxial extensiometry

After the OCE measurements, sclera and cornea strips were extracted from the globes and mechanically stretched. The epithelium, choroid and retina were removed, and samples were stored less than 60 min in a custom-made humid chamber with salt solution before tensile testing. Sclera strips (3 mm × 20 mm) were cut in meridional direction, around the SN and IN locations (see Fig. 1d). Corneal strips (3 mm × 20 mm) were cut along the superior-inferior direction. The dimensions of all strips were measured with a caliper (analogue, Alca, 0.05 mm, 1–150 mm) and a micrometer (digital, Mitutoyo, model 293-240-30).

Uniaxial tensile test was performed using a UStretch (CellScale, Waterloo, ON, Canada). The free length between the clamps was 6 mm before stretching. Specimens were subjected to five loading/unloading cycles at a rate of 1.0 mm/min with a preload force of 0.05N during the first cycle only. During the cycles, the specimens were kept immersed in saline solution. All specimens were cut and tested immediately after the OCE measurements. Stress–strain curves after five preconditioning cycles were used for stiffness estimation. Young’s modulus was obtained from the data of these curves at 7% of strain. This strain was calculated for IOP = 15 mmHg (see also detailed explanation in Supplementary section A).

### Statistical analysis

Statistical analysis was performed using IBM SPSS (Version 27.0). Data were labeled and compared between locations (C, SN, IN, ST, IT) and zones (anterior, posterior, meridional, circumferential). ANOVAs were conducted to assess differences between the means of the parametric variables: phase speed, maxMPAC and MAA. Non-parametric Kruskal–Wallis H tests were used to compare differences between the means of the variables: shear modulus, thickness, Young’s modulus, and NFA. Specific location and zone effects were evaluated using a post-hoc test with a Bonferroni correction. Significance was set at p-value of 0.05. All values in bar plots are indicated as mean ± standard deviation and the boxplots display the first quartile, median, and third quartile. Error bars in boxplots are the 95% confidence interval.

## Results

### Phase speed at different eye globe locations

**Fig. 3:**
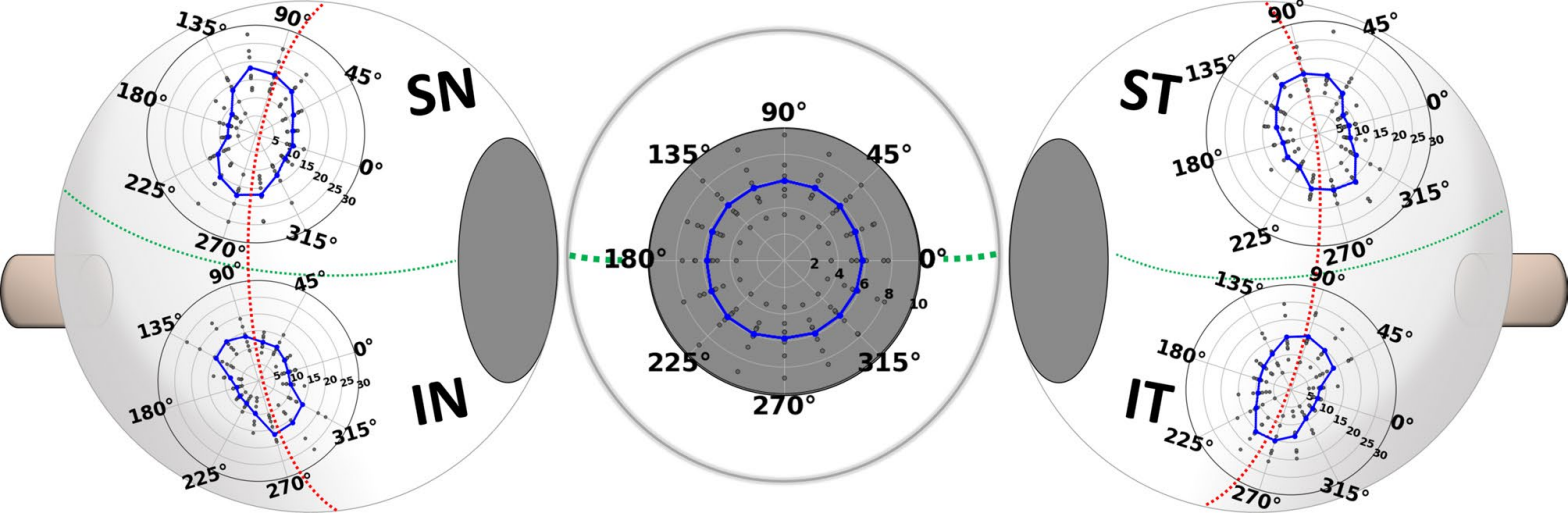
Phase speed polar plots across different ocular globe regions*.* Above polar plots show values of phase speed (in m/s) as function of wave propagation angle measured by the ACUS-OCE method in the sclera (left: superior-nasal (SN) and inferior-nasal (IN) locations; right: superior-temporal (ST) and inferior-temporal (IT) locations; center: cornea). The dark blue line shows the mean value of phase speed at every angle of all samples at a constant IOP = 15 mmHg, data points are shown as gray dots. Note that the scale in the central graph (cornea) is different from the neighboring graphs (sclera) with scale units being 2 m/s for the cornea and 10 m/s for the sclera.

Figure 3 provides an overview of the phase speed polar plots for each ocular location. A visual inspection of Fig. 3 shows that the direction of fastest phase speed tends to align with the equatorial circumferential axis (red, dashed line in Fig. 3) and appears to be horizontally symmetric, while the cornea follows a more circular symmetric distribution, which suggests that the rabbit sclera is anisotropic, showing a preferential orientation. This orientation is not influenced by the positioning of the eye (see Supplementary Fig. 1).

An exploratory estimation of these symmetries was performed using only the vertical and horizontal axes of the phase speed polar plots, in both sclera and cornea. Supplementary Table 1 presents the results of the mean phase speed at these two specific axes for the five locations. A V-H ratio was defined by dividing the mean vertical speed by the mean horizontal speed. For all samples, the calculated ratios suggest a more circular symmetric behavior of the cornea with a calculated V-H ratio of 1, in contrast to a more horizontally symmetrical behavior (“earing”) of the sclera with a V-H ratio close to 2.

### Phase speed, shear modulus and thickness estimates

**Fig. 4:**
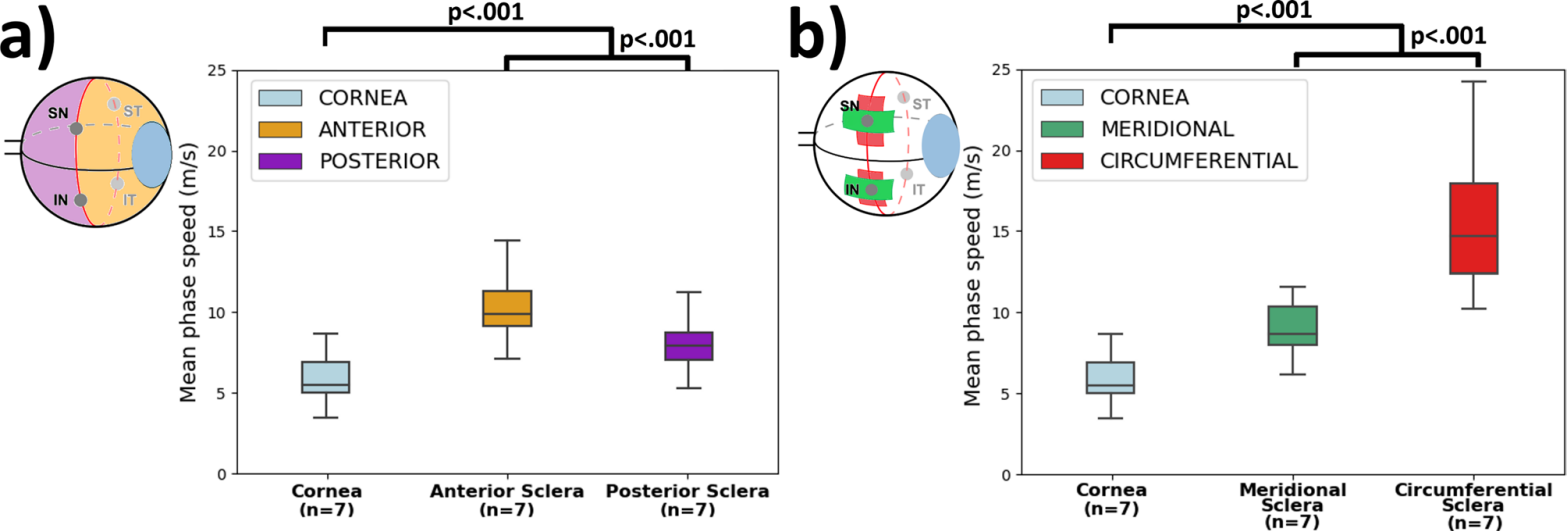
The phase speed depends on the scleral zone. Shown here is the mean phase speed of the cornea (light blue) in comparison to **(a)** the anterior (orange) and posterior (purple) scleral zone and **(b)** the meridional (green) and circumferential (red) zone. The mean scleral phase speed was calculated from the phase speeds at the four investigated locations (SN, IN, ST, IT).

Phase speed, shear modulus, and thickness of the sclera and cornea were determined for each location and semi-axis angle. Figure 4 shows the mean phase speed distributed by zones. Comparison of corneal and scleral data revealed that the average wave propagated significantly slower (p < 0.001) in the corneal tissue (5.9 ± 1.4 m/s, light blue bars) than in the scleral tissue (12.1 ± 3.2 m/s). On the other hand, in the scleral tissue the wave propagated significantly faster (p < 0.001) in the anterior than the posterior globe (10.1 ± 1.8 m/s vs. 8.0 ± 1.7 m/s, Fig. 4a), and in the circumferential than in meridional directions (15.4 ± 3.7 m/s vs. 9.1 ± 1.5 m/s, Fig. 4b). All phase speed values are shown in Supplementary Table 2.

**Fig. 5:**
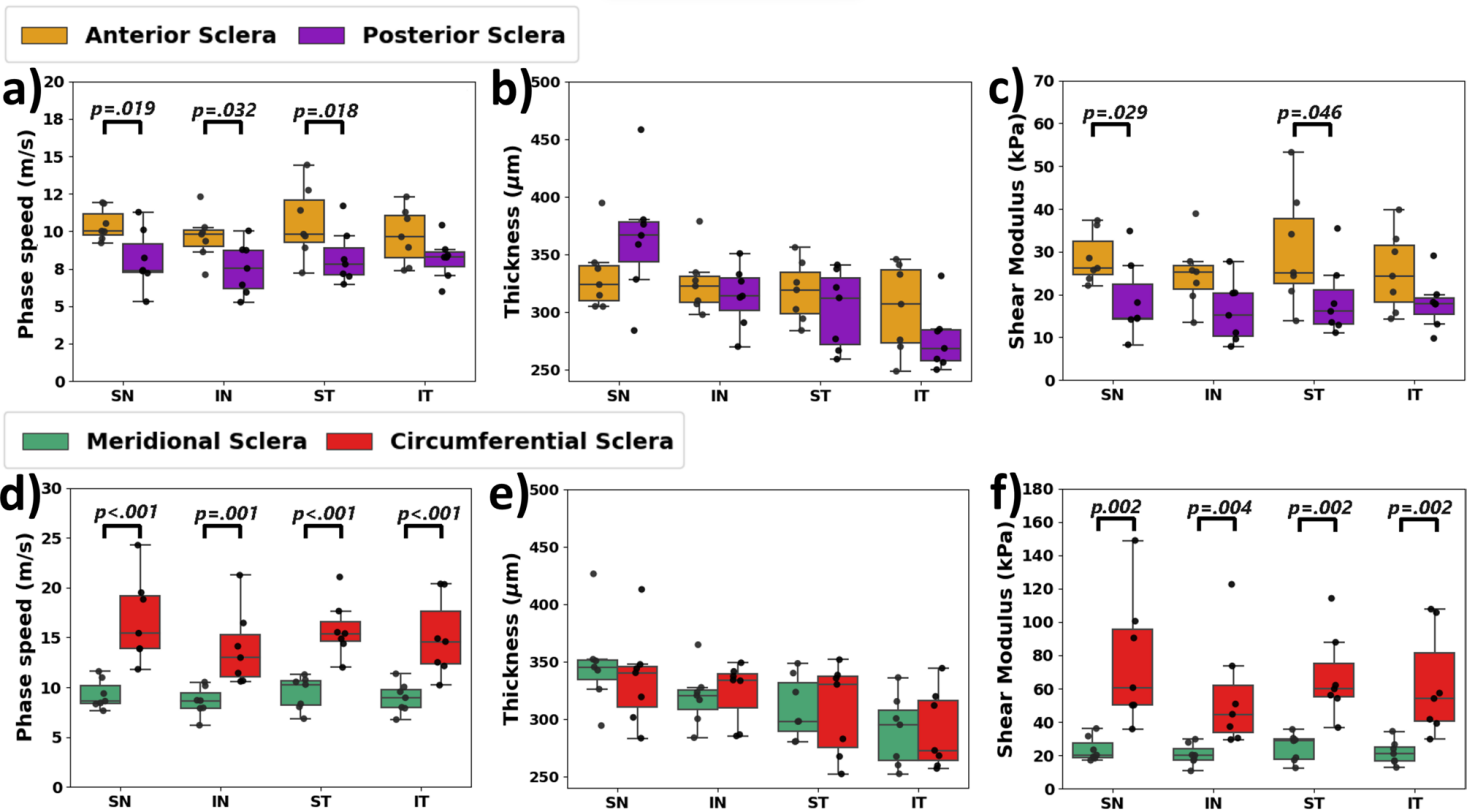
Comparison between measurements at each scleral location. Box plots of phase speed **(a,d)**, thickness **(b,e),** and shear modulus **(c,f)** are shown for all seven rabbit eyes at 15 mmHg. Scleral measurements showed in the anterior (orange), and posterior (purple) zones (**a–c**), and along meridional (green) and circumferential (red) zones (**d–f**) at each tested location (SN, IN, ST and IT). The line inside the box is the median, and data points are shown as black dots. p-values are shown in the graph only for significant differences.

Figure 5 shows the results of the phase speed (Fig. 5a, d), sample thickness (Fig. 5b, e), and shear modulus (Fig. 5c, f) for the anterior (orange bars), posterior (purple bars), meridional (green bars) and the circumferential (red bars) zones of the sclera, and for all scleral locations (SN, IN, ST, IT, see Fig. 1a) individually. Figure 5a shows significantly (p ≤ 0.032) higher phase speeds in the anterior zones with respect to the posterior zones at the following locations: SN, IN and ST (see Supplementary Table 2). In addition, phase speed measurements along circumferential direction were significantly higher (p = 0.001) than in meridional direction at all locations (see Fig. 5d).

Scleral thicknesses were determined at each location. Pairwise comparisons indicated that scleral thickness (316.8 ± 39.8 μm) was significantly lower (p < 0.001) than corneal thickness (511.1 ± 58.9 μm). Scleral thickness was greatest in posterior/meridional scleral location in SN (364.5 ± 53.2 μm/348.1 ± 39.9 μm), and lowest in posterior/meridional scleral location in IT (276.2 ± 27.7 μm/289.5 ± 30.9 μm).

The shear modulus was estimated from the mRLFE model using the measured phase speed and thickness data from each semi-axis. Statistical analysis showed that the shear modulus of the anterior sclera (27.3 ± 1.8 kPa) was significantly higher (p < 0.001) than that of the posterior sclera (17.8 ± 1.4 kPa), and cornea (11.2 ± 4.1 kPa) (see Supplementary Table 2). In the subsequent analysis by locations, the shear modulus was significantly higher (p ≤ 0.046) in the anterior superior than in the posterior superior zone (SN and ST in Fig. 5c). The mean shear modulus in the circumferential direction (65.4 ± 31.9 kPa) was significantly higher (p ≤ 0.004) than in the meridional direction (22.5 ± 7.2 kPa) at all scleral locations (Fig. 5f and Supplementary Table 3).

### Phase speed anisotropy

**Fig. 6:**
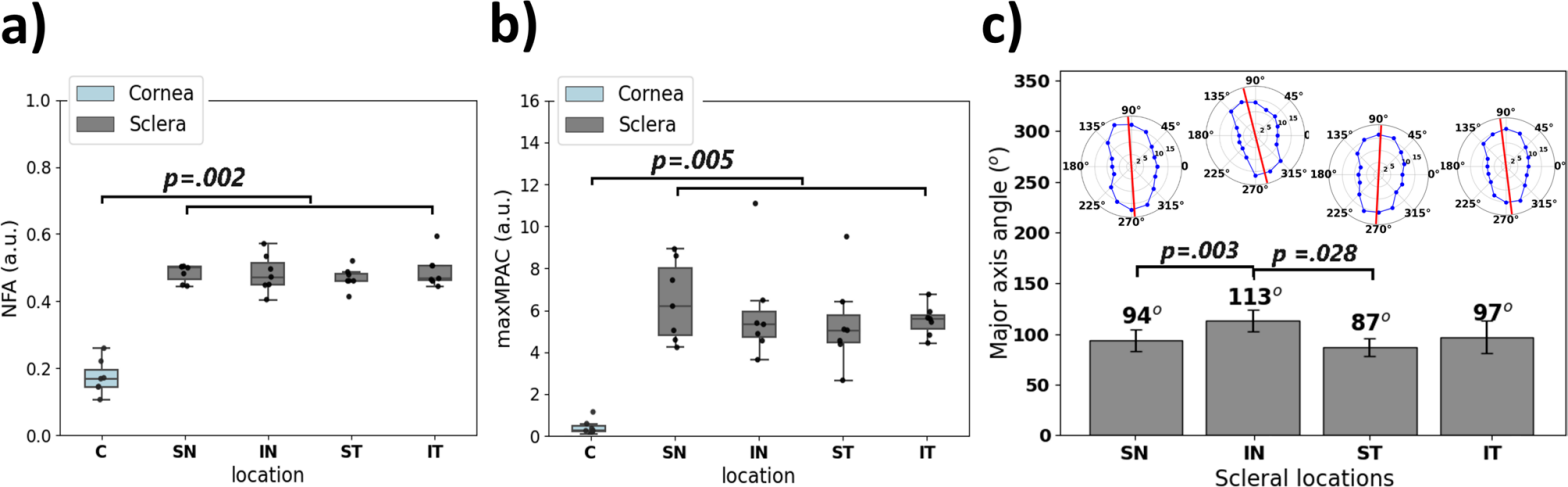
Phase speed anisotropy at different ocular locations*.* Shown here is (**a)** the normalized fractional anisotropy (NFA), (**b)** the maximum value of the modified planar anisotropy coefficient (maxMPAC) for the cornea and the sclera; and (**c)** the major-axis angle (MAA) for scleral locations only. The polar plot of the mean phase speed is shown at the top of each bar, where the red lines represent the preferred orientation at the corresponding location. MAA was measured from the corneal limbus to the optic nerve. p-values are shown only for significant variations.

Figure 6 shows the calculated anisotropy parameters NFA (Fig. 6a), maxMPAC (Fig. 6b), and the MAA (Fig. 6c). Both NFA and maxMPAC showed a significant difference (p ≤ 0.005) in anisotropy between the cornea (0.17 ± 0.05 and 0.42 ± 0.33, respectively) and the sclera (0.48 ± 0.04 and 4.73 ± 2.71, respectively), with the sclera being more anisotropic by a factor of 3 and 11, for NFA and maxMPAC, respectively. There was no significant difference between scleral locations (p = 0.94). In addition, the angle-dependent MPAC is presented as a function of each directional angle to exhibit differences in corneal and scleral locations (see Supplementary Fig. 2). Figure 6c shows the major axis angle (MAA), which represents the direction of the first principal axis in the polar plots, for all scleral locations. The MAA shows a subtle tilt from the equatorial circumferential axis (90°, 270°), which (considered the orientation at all locations, see Fig. 3) can be interpreted as a tilt towards the optic nerve head for location SN, IN, and IT, and towards the corneal limbus for location ST (see Supplementary Fig. 3). Specifically, the major axis angles at the lower eye locations (IN and IT) had a greater inclination, with the mean angle being greater at the IN location than at the IT location (113.2° ± 9.9° vs. 97.0° ± 14.6°). The orientation in superior locations was similar (93.9° ± 10.0° and 87.0° ± 8.3°, for SN and ST, respectively).

### Comparison of shear modulus and Young’s modulus

**Fig. 7:**
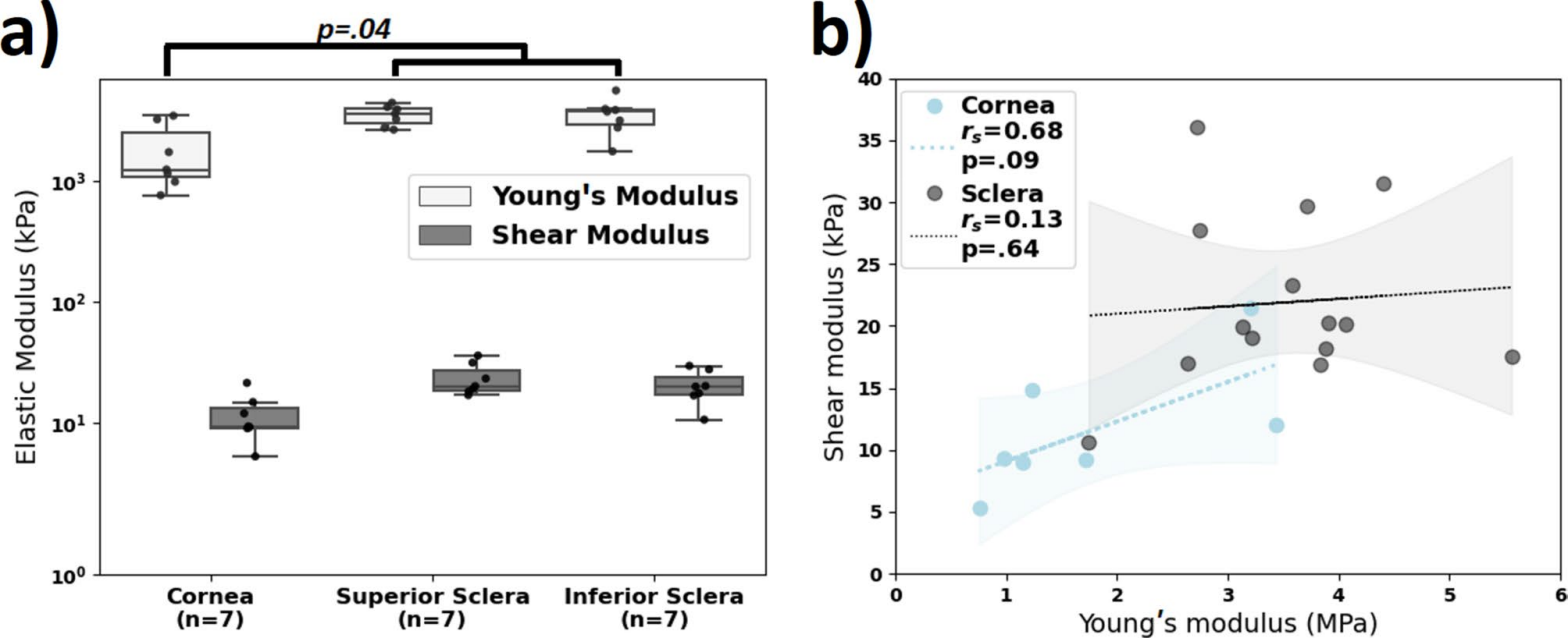
Comparison of the Young’s modulus and the shear modulus of the cornea and nasal sclera. **(a)** Young’s modulus (white) at 7% strain and shear modulus (gray) at predefined locations (cornea, superior and inferior sclera) represented as boxplots. The y-axis scale is logarithmic. Black dots indicate data points, and p-values are given for significant differences between respective groups. (**b**) Scatterplot (Young’s vs. shear modulus) for the cornea (light blue) and the sclera (gray). Dashed lines represent the corresponding linear regressions, and shaded areas represent 95% of confidence interval, for corneal and scleral data. Spearman correlation coefficient r_s_ and associated p-values are shown in the upper left corner.

The shear modulus (SM) estimated from OCE measurements was compared to the Young´s modulus (YM) obtained from uniaxial extensiometry. The SM values were taken at the corresponding meridional axes (see Fig. 5f and Supplementary Table 3) for the scleral nasal locations (SN, IN), and on the vertical axes (at 90° and 270°) of the cornea, to match with the orientation of the strips used in the tensile test (Fig. 1d). Figure 7a shows that corneal SM and YM (11.6 ± 4.8 kPa and 1.8 ± 0.7 MPa, respectively) were significantly lower (p = 0.04) than scleral SM and YM (22.0 ± 6.6 kPa and 3.5 ± 0.9 MPa, respectively). Using Spearman correlation analysis, SM and YM showed a moderate correlation in the cornea, without reaching statistical significance (${r}_{s}$ = 0.68, p = 0.09), and no correlation in the sclera (${r}_{s}$ = 0.13, p = 0.64) (see Fig. 7b).

## Discussion

We have presented, for the first time to our knowledge, multi-directional wave-based Optical Coherence Elastography as a method to map the anisotropic behavior across the sclera in comparison to the cornea, in excised (whole globe) rabbit eyes. Both the Young’s modulus and the shear modulus were lower in the cornea than in the sclera (with the YM two orders of magnitude higher than the SM, Fig. 7a), and while in the cornea there was some association (not reaching statistical significance) between moduli, they appeared uncorrelated in the sclera. We also found that the sclera is stiffer, both circumferentially (along the equator) and in the anterior zone, than meridionally, and in the posterior zone, respectively. The highest shear modulus values were found in the circumferential superior sclera (Fig. 5c, f). Multi-directional OCE allowed a radial mapping of mechanical properties in different locations and orientations on intact eyes. Furthermore, the shear modulus obtained from OCE may be capturing highly relevant information related to biochemical composition and collagen organization and interweaving.

The Young’s modulus describes tensile elasticity, quantifying the relationship between stress and strain under uniaxial force, compression, or tension. In contrast, the shear modulus describes the tendency of an object to deform in response to a tangential force, causing a change in shape without a change in volume. The lack of correspondence between Young’s and shear modulus is not unexpected, as simple relations only hold for isotropic homogeneous materials, while the cornea and sclera material properties rather respond to a structural composite material with high degree of heterogeneity and anisotropy^14,46,58^, which we found to be even larger in the sclera.

Differences in Young’s and shear modulus have been reported in the cornea as a function of age with the former doubling over an 80-year span^59^ and the latter remaining constant over an age range of 25 to 67 years^29^. These discrepancies are explained by the fact that the two techniques address distinct mechanical properties which may vary depending on physiological conditions or disease. However, the nearly-incompressible transverse isotropy (NITI) model^46^ has demonstrated a decoupling between tensile and shear response in porcine corneal OCE measurements, showing that corneal biomechanics describe an isotropic behavior at pressures below 30 mmHg. In particular, it has been argued that extensiometry may be associated with the stiffness of the individual collagen fibrils, whereas the OCE technique may depend primarily on the properties of the interfibrillar and interlamellar matrix. This is of a special interest in the sclera, where regional variations in the composition of the fibrillary collagen network and GAGs have been linked to scleral mechanical behavior^12,60^.

In addition, a study of scleral elastic response to IOP variations using a constitutive model^22^ showed that the elastic modulus has significant effect on collagen fibril strain, while the shear modulus has impact on the deformation of the scleral shell plane. The notion that the shear modulus estimated from OCE may target the mechanical properties of scleral extracellular hydrogel and stiffness resulting from collagen fibril interweaving directly, may partly explain the lack of significant differences in the elastic behavior of GAG-depleted and normal scleral strips in a uniaxial testing protocol^12^, despite their fundamental role on the regulation of collagen assembly and organization^61^.

Corneal and scleral tissue have different mechanical properties which are influenced by their specific composition and organization of collagen fibrils^1^. Human corneal collagen fibrils have a small diameter (25 nm) and are moderately organized (parallel lamellae of collagen fibrils), compared to scleral collagen, which has wider fibrils (ranging from 25 to 230 nm) and an interweaved structure (non-uniform and intertwined collagen fibril bundles)^2^. These differences in the internal organization of the tissue are likely behind the variations in phase speed (and consequently, the shear modulus) between the corneal (5.9 ± 1.4 m/s) and scleral tissue (12.1 ± 3.2 m/s) reported here. In addition, our average data are in good agreement with recent results in patient studies that reported a wave speed twice as high in the sclera than in the cornea^29^.

The relatively lower anisotropy of the cornea compared to the sclera is consistent with previous work using different techniques to probe corneal mechanical properties. For example, angle-dependent phase speed measurements in corneal tissues have been studied with Brillouin microscopy and constitutive models^36,37^ to relate the Brillouin modulus to elastic anisotropy. Eltony et al*.*^37^ estimated a degree of anisotropy considering the aligned of the fiber composite in the cornea reporting values of 0.18 ± 0.03 (porcine) and 0.12 ± 0.02 (human). For better comparison, we calculated an analogous anisotropy parameter (Supplementary Fig. 4) based on phase speed^62^, which resulted in lower results for the rabbit cornea (0.03 ± 0.11), and higher ones in the sclera (0.78 ± 0.22) in comparison to the anisotropy calculated by Eltony et al*.*^37^.

In this work, the phase speed was found to be dependent on the scleral zone and direction. Local measurements in the sclera indicated significantly higher average phase speeds in the anterior zone (10.1 ± 1.8 m/s) than in the posterior zone (8.0 ± 1.7 m/s) and almost double along circumferential direction (15.4 ± 3.7 m/s) than in meridional direction (9.1 ± 1.5 m/s). Earlier literature using piezoelectric excitation reported similar values of phase speed in porcine^43,52^ and human^29,43^ sclera. Specifically, the phase speed was at least 63% faster in the circumferential zones than in other areas of the scleral tissue. This finding seems to be consistent with other research^22,30–32^ which found that this direction-dependent mechanical behavior is associated to the preferential orientation of the collagen fibers. We also found a comparable shape between the collagen fibril distribution maps reported in previous studies^22,31,63^ and the phase speed polar plots at the same scleral location in the current study.

To study anisotropy, we defined three parameters: normalized fractional anisotropy, maximum value of modified planar anisotropy coefficient and major-axis angle to compared cornea/sclera anisotropy with data from previous studies. We found significantly higher anisotropy in the sclera than in the cornea for NFA and maxMPAC parameters, however, no significant differences in anisotropy were found between scleral locations. The apparent corneal isotropy, which has been extensively studied in previous studies^64–66^, was not examined in this report because the focus was on the sclera. But, we should highlight that the NFA value (0.17 ± 0.05) in rabbit corneal tissue was similar to that reported previously (0.56 ± 0.05) in porcine corneas^42^, and that polar plots of MPAC values (Supplementary Fig. 2) also showed identical anisotropic mechanical pattern in corneas at 15 mmHg. Moreover, the MAA, which possibly reflects the predominant collagen fibril orientation in the sclera, showed a dorsal tilt (towards the optic nerve head) for all locations except at the ST location where it was tilted slightly ventrally (Fig. 3 and Supplementary Fig. 3).

Anterior and posterior scleral zones presented different elastic behavior. We found that the shear modulus in the anterior scleral zone was 53% higher than in the posterior superior zone. These differences are consistent with results from studies using tensile tests, which found that the tangent modulus of the anterior sclera was twice that of the posterior sclera^18,67^. Prior work interpreted those differences in mechanical properties as arising from regional variations in scleral thickness for humans^18,19^ and rabbit eyes^68^. However, our OCE-based results show that, when the scleral thickness is compensated for, the posterior scleral shear modulus is still lower than that of the anterior sclera, at all locations. Interestingly, in the SN region, scleral thickness is higher than in other locations, however wave speed is still lower than in anterior regions, indicating that it is the inherent mechanical properties, and not scleral thickness, which make the posterior sclera more susceptible to deformation.

The regional variations in shear modulus between anterior and posterior sclera may be behind the preferential axial elongation of the eye during emmetropization. These differences are likely enlarged following myopigenic signaling, which leads to further scleral remodeling in the posterior sclera, and myopia development.

The highest shear modulus in the rabbit sclera was measured at circumferential orientation along the equator. On average, the circumferential shear modulus was 66% higher than that of the meridional sclera at all locations. Our results are consistent with those observed in earlier studies in which the circumferential direction was reported to be stiffer than the meridional direction after biaxial mechanical testing in the porcine sclera^21,33^. They also confirm a preferential orientation and suggest a high density and/or high degree of interweaving of collagen fibrils in the circumferential direction at the equator. However, our results differ from the earlier published preferential meridional orientation for rat scleral fibers^19^, but they are broadly consistent with earlier experiments^31,32,63^ and constitutive models^22^ in mouse, human, and rat scleras.

The findings in this work are subject to at least three limitations. First, scleral tissue thickness, which is thicker than the investigated rabbit tissue (500–800 µm in humans vs. 200–400 µm in rabbits), may be difficult to estimate from OCT images due to higher scattering and opacity of the sclera compared to the cornea. The custom-developed PhS-SS-OCT system uses 1300 nm swept-source and has a high penetration because of the long wavelength band and ideally signal roll-off along the axial depth, making it challenging to improve the imaging capabilities for the sclera. Second, the shear modulus was calculated based on a linear elastic approximation. The modified Rayleigh-Lamb frequency equation model considers a viscoelastic material with isotropic and homogenous response. Nevertheless, the sclera is a non-linear elastic material^22^ with an anisotropic structure^19^ and subject to prestress tension^69^. These are important considerations for modeling wave propagation in the sclera in future research. In this study, our calculated values of the shear modulus in the cornea agree with previous studies in rabbits using atomic force microscopy^70^ and an inflation test with inverse analysis^71^, so it is likely that these assumptions only moderately affect our results. In any case, given the expected intrinsic differences between Young’s and shear modulus in the sclera, future work may elucidate the physicochemical and microstructural factors underlying the scleral biomechanical properties captured by each technique, and the value that they offer in understanding basic mechanisms of the scleral physiology, its changes with disease and its response to treatment.

Third, an important consideration regarding the elastic properties of the sclera is the influence of the fixed IOP during OCE measurements. IOP induces a pre-stressing effect on ocular tissues that directly affects tissue stiffness^25,72^. Simulations have shown that the estimated phase speed at frequencies around 2 kHz (the frequency used in this manuscript to estimate the shear modulus) can be affected by pre-stress^69^ in a transversely isotropic material. Specifically, Sun et al.^73^ have described a two-fold reduction in corneal shear modulus at 15 mmHg in porcine eyes, which may also apply to the sclera. Thus, although OCE has a key advantage over strip extensiometry in its non-invasive nature, this technique requires the eye to be maintained at constant IOP conditions, or it could have a significant effect on the shear modulus estimation. Therefore, future work using our set-up will include the measurement of controlled IOP changes and its effects on the estimation of scleral biomechanical parameters. Favorably, OCE at lower frequencies (1–3 kHz) appears to have the advantage of introducing less additional stress^69^ than other non-invasive techniques, such as air-puff deformation.

In summary, we have shown that multi-directional ACUS-OCE is an effective, non-invasive, and non-contact technique to estimate the biomechanics of the sclera. In this study, the biomechanics of the sclera of intact rabbit eyes were determined in different zones (anterior, posterior, meridional and circumferential) and orientations (16 angles) using a single measurement. The multi-directional detection of wave propagation in different regions provides a unique opportunity to map the sclera and to obtain its mechanical anisotropy. These results are expected to find application in the development of improved computational or physical ocular models to study scleral involvement in healthy eyes, as well as to evaluate the effects of myopia-triggering signals and identify appropriate biomarkers for disease monitoring. ACUS-OCE may also assist in evaluating possible myopia treatment strategies to strengthen the sclera.

## Supplementary Information

Supplementary Information.

## Data Availability

The datasets generated during and/or analyzed during the current study are available from the corresponding author on reasonable request.
