# Supplementary material for: Revealing regional variations in scleral shear modulus in a rabbit eye model using multi-directional ultrasound optical coherence elastography
Source: Sci Rep. 2024 Sep 9;14:21010. doi: 10.1038/s41598-024-71343-0 (PMC11384758; doi:10.1038/s41598-024-71343-0)
Supplement: Supplementary file 1 — Supplementary Information. [file 41598_2024_71343_MOESM1_ESM.pdf]

Revealing regional variations in scleral shear modulus in a rabbit eye model using multi-directional ultrasound optical coherence elastography

Lupe Villegas[1], Fernando Zvietcovich[2], Susana Marcos[1,3], Judith S. Birkenfeld[1]
[1] Instituto de Óptica, Consejo Superior de Investigaciones Científicas, Madrid, Spain
[2] Department of Engineering, Pontificia Universidad Católica del Peru, Lima, Peru
[3] The Center for Visual Science, Rochester, NY, United States

Corresponding authors: lupe.villegas@io.cfmac.csic.es, j.birkenfeld@io.cfmac.csic.es

Supplementary Data

A. Relationship between intraocular pressure and strain

To compare shear modulus getting from rabbit eyes under an IOP of 15mmHg with samples gotten from the same eyes and locations, the relationships between IOP and stress-strain was determined by approximations. The sclera in rabbit eyes can be approximately as spherical shell of radius r and thickness t filled with an aqueous liquid. The tension σ over the sclera due to the internal surface pressure (in this case IOP) can be estimated using the Young–Laplace equation: $\sigma = \frac{P\,r}{2t}$. On the other hand, specimens exhibit exponential stress-strain behavior ($\sigma - \varepsilon$) from tensile test was estimated by $\sigma = A(e^{B\varepsilon} - 1)$ [1] where A and B are constants so Young's modulus can be approximated by $E = \frac{d\sigma}{d\varepsilon} \approx AB(e^{B\varepsilon})$. Considering the same applied external force and joining the equations, it got $\frac{P\,r}{2t} \approx AB\varepsilon(1 + B\varepsilon)$ .The parameters used are $r = 8.12mm$, $t = 300\ \mu m$, $P = 15mmHg$ [2] and A=.0008, B=80.0 MPa from our calculated stress-strain curves for superior nasal sclera. It was estimated at a strain of 7% for 15mmHg.

B. Influence of rotation in speed anisotropy

The influence of the eye rotation on the polar plot was specifically examined using one eye. The eye was rotated 90 degrees counterclockwise, and excitation was applied at the same location. The following Fig. 1 shows the resulting mean speed propagation in the polar tracing of the unrotated and rotated eye. The speed values are almost superimposed in Fig. 1.c.

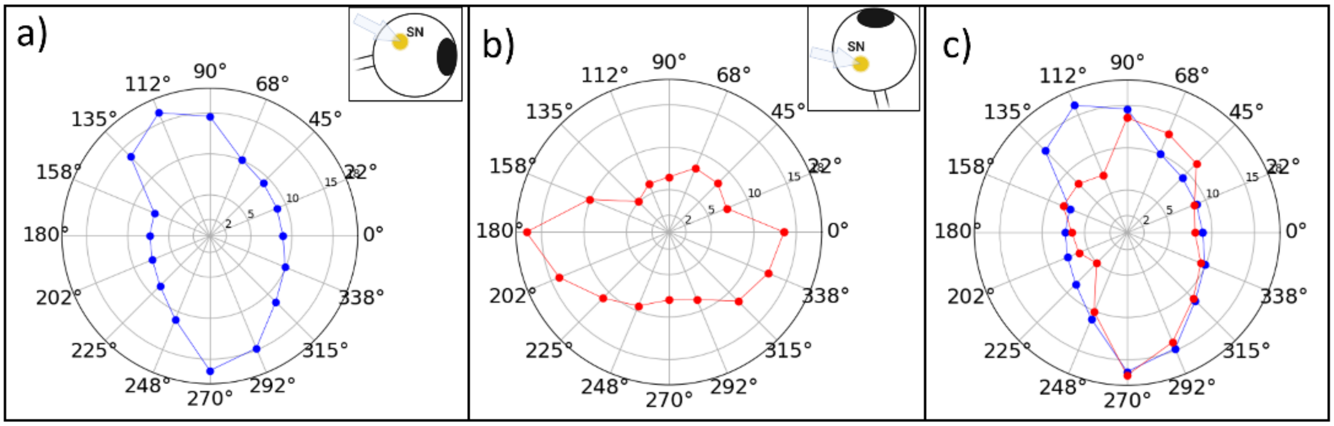

Fig. 1. ***The anisotropic effect is not influenced by the technique.*** Mean values of phase speed as function of propagation angle measured by OCE in the same eye at the same scleral point (SN) before (a) and after (b) 90 degrees rotation. c) Both plots are combined on the same axes.

C. Tables of measurements of vertical-horizontal ratio, phase speed, shear modulus, thickness and Young's modulus values for eyeball zones

	Location	Horizontal Axis		Vertical Axis		Vertical-horizontal ratio
		0º	180º	90º	270º	
Mean phase speed in cornea (m/s)	C	5.80±1.42	5.75±1.32	6.04±1.91	5.83±1.73	1.0
Mean phase speed in sclera (m/s)	SN	10.64±1.58	7.89±1.63	17.15±4.41	17.59±4.23	1.9
	IN	9.27±1.66	6.86±1.16	13.90±3.45	16.73±7.03	1.9
	ST	8.30±1.64	9.83±1.95	16.52±4.50	15.53±2.33	1.8
	IT	7.91±0.99	9.48±1.17	16.04±5.15	15.21±4.80	1.8

Table 1. Mean phase speed (m/s) for cornea and sclera at locations in the horizontal and vertical axis of wave propagation. The mean phase speed with standard deviation is shown for horizontal and vertical axes at different locations. Values of the vertical-horizontal ratio close to 1 indicate a more circular symmetric behavior.

The mean values and confidence intervals of the measurements are summarized by zone and location in Table 1 and Table 2.

Zone	Location	Phase Speed (m/s)	Thickness (µm)	Shear Modulus (kPa)
**Cornea**	**C**	† 5.9 (5.7-7.0)	‡ 511.1(487.9-534.4)	† 11.2 (9.6-12.9)
**Anterior sclera**	**SN**	10.4 (9.1-11.8)	331.8(305.8-357.8)	28.5 (21.9-35.1)
	**IN**	9.6 (8.3-11.0)	325.3(299.3-351.2)	24.7 (18.1-31.4)
	**ST**	10.6 (9.3-11.9)	317.7(291.7-343.7)	30.4 (23.8-37.0)
	**IT**	9.7 (8.3-11.1)	302.9(276.9-328.9)	25.4 (18.7-32.0)
	Total	***†10.1(9.4-10.8)***	***‡ 319.4(307.3-331.5)***	***†27.3 (23.7-30.9)***
**Posterior sclera**	**SN**	8.1 (6.8- 9.5)	364.5(338.5-390.5)	18.7 (12.1-25.3)
	**IN**	7.5 (6.2- 8.9)	313.9(287.9-339.9)	16.0 (9.4-22.6)
	**ST**	8.3 (6.9- 9.6)	302.0(276.0-327.9)	18.7 (12.1-25.4)
	**IT**	8.2 (6.8- 9.5)	276.2(250.2-302.2)	17.9 (11.3-24.6)
	Total	***†8.0 (7.4-8.7)***	***‡ 314.1(295.6-332.7)***	***†17.8 (15.0-20.7)***

Table 2. Mean values (and 95% confidence intervals) of phase speed, thickness and shear modulus for three zones: cornea (C), anterior sclera (AS) and posterior sclera (PS). Multiple comparisons between zones revealed statistically significant differences shown between labeled values (†: $p<.004$). Only C vs. AS and C vs. PS comparisons in tissue thickness are significant (‡: $p<.001$).

Zone	Location	Phase Speed (m/s)	Thickness (µm)	Shear Modulus (kPa)	Young's Modulus at 7% of strain (MPa)
**Cornea**	**C**	† 5.9 (5.7-7.0)	‡511.1(487.9-534.4)	† 11.2 (9.6-12.9)	1.8 (0.8 - 2.8)
**Meridional sclera**	**SN**	9.3 (7.1 -11.5)	348.1 (322.5-373.8)	23.6 (5.5-41.7)	3.5 (2.9-4.1)
	**IN**	8.6 (6.4 -10.8)	319.6 (293.9-345.2)	20.4 (2.3-38.5)	3.5 (2.4 -4.6)
	**ST**	9.4 (7.2 -11.6)	309.8 (284.2-335.5)	24.6 (6.5-42.7)	
	**IT**	8.9 (6.7 -11.1)	289.5 (263.9-315.2)	21.7 (3.6-39.8)	
	Total	†***9.1 (7.9-10.1)***	‡***316.8 (299.5-334.0)***	†***22.5 (19.7-25.3)***	
**Circumferential sclera**	**SN**	16.8 (14.6-19.0)	335.5 (309.9-361.2)	76.6 (58.5-94.7)	
	**IN**	13.9 (11.7-16.1)	323.8 (298.2-349.5)	55.5 (37.4-73.6)	
	**ST**	15.8 (13.6-18.0)	308.3 (282.6-333.9)	67.2 (49.1-85.3)	
	**IT**	15.0 (12.8-17.2)	290.5 (264.8-316.1)	62.1 (44.0-80.3)	
	Total	†***15.4 (14.3-16.5)***	‡***314.5 (297.3-331.8)***	†***65.4 (53.0-77.8)***	

Table 3. Mean values (and 95% confidence intervals) of phase speed, thickness, shear modulus and Young's modulus for zones: cornea (C), meridional sclera (MS) and circumferential sclera (CS). Multiple comparisons between zones revealed statistically significant differences shown between labeled values (†: p<.001). Only C vs. MS and C vs. CS comparisons in tissue thickness are significant (‡: p<.001).

D. Angle-dependent modified planar anisotropy coefficient

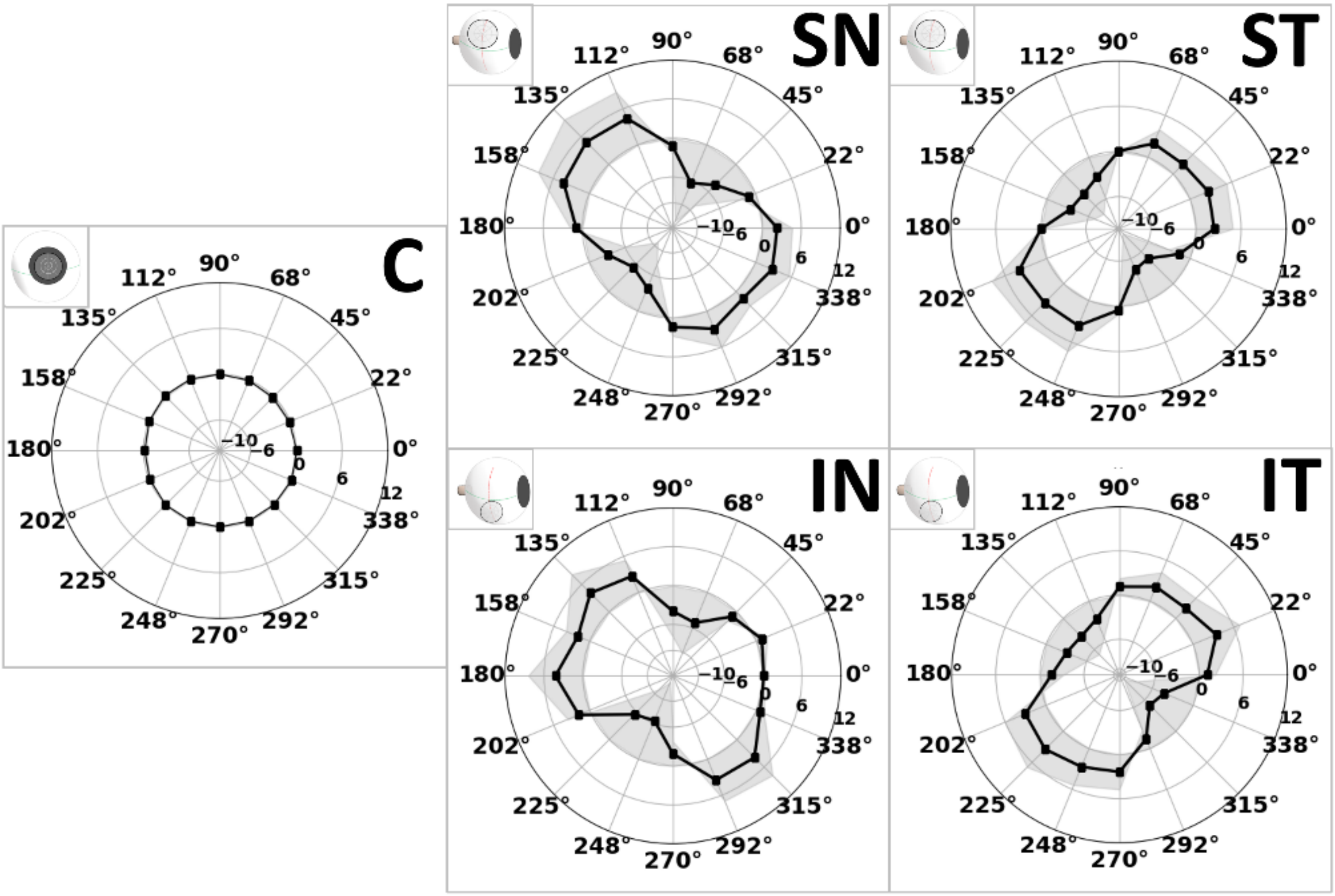

Fig. 2. ***Modified planar anisotropy coefficient for corneal and scleral locations.*** Polar plots of calculated values of MPAC (u.a.) for each direction (16 semi-axes in total) for cornea (C) and sclera (above: superior-nasal (SN) and superior-temporal (ST) locations; below: inferior-nasal (IN) and inferior-temporal (IT) locations). Black line indicates the average values for 7 eyes at each directional angle. The standard deviation is represented as shaded areas in gray. The location on the eye is represented by a small icon in the upper left corner.

E. Major-axis angle variations respect to the equatorial circumferential axis

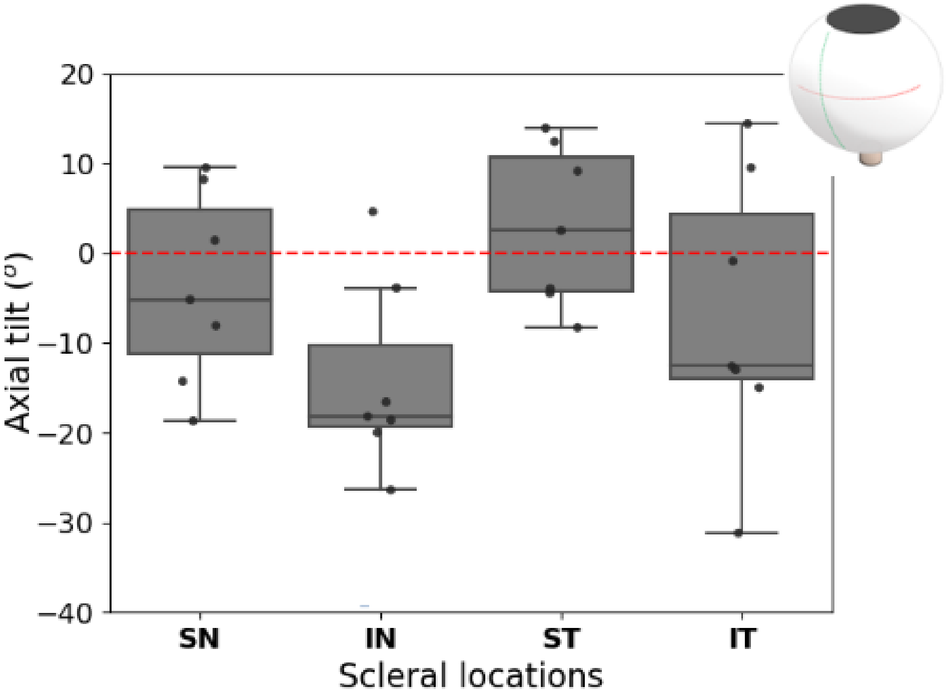

Fig. 3. ***Major-axis angle tilt for scleral locations.*** The boxplot of variation between MAA and the equatorial circumferential axis (90 degrees) are shown. Red line indicates the zero value. Positive values indicate that major axis is tilted towards the corneal limbus. Negative values indicate that major axis is tilted towards the optic nerve. Median values of MAA variation are: -5.2^{o}(SN), -18.2^{o}(IN), 2.5^{o}(ST), -12.6^{o}(IT). Error bars in plots are the 95% confidence interval, the bottom and top of the box are the 25th and 75th percentiles, the line inside the box is the median, and data points are shown as black circles.

F. Anisotropic parameter of Thomsen

Thomsen [3] defined an anisotropic parameter based in wavefront propagation in a homogeneous anisotropic elastic medium. In our case, we adapted the P-wave anisotropy parameter as the fractional ϵ_L difference between vertical (fast) and horizontal (slow) phase speed as:

$$\epsilon_L = \frac{c_{L_{90^\circ}} - c_{L_{0^\circ}}}{c_{L_{0^\circ}}} \quad \text{(F.1)}$$

where $c_{L_{0^\circ}}$ and $c_{L_{90^\circ}}$ is the wave propagation speed at 0^0 and 90 orientation angles, respectively. Values of the parameter $\epsilon_L < 0.2$ are considered as a weak-to-moderate anisotropy. The results in Fig. 4. show that the degree of anisotropy was significantly higher (p=.04) for scleral (0.78±0.22) than for corneal tissue (0.03±0.11).

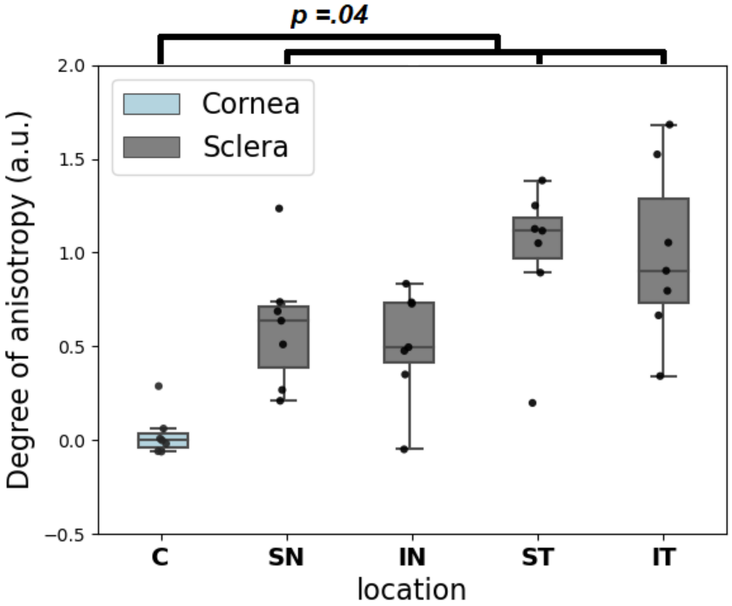

Fig. 4. ***Degree of anisotropy for corneal and scleral locations.*** The boxplots show of the degree of anisotropy for the cornea (C) and in the nasal and temporal sclera (SN, IN, ST, IT). Error bars in plots are the 95% confidence interval, the bottom and top of the box are the 25th and 75th percentiles, the line inside the box is the median, and data points are shown as black circles.

G. Anisotropic equations

The phase speed of the Lamb wave speed c_L was quantified at both corneal and scleral locations. The normalized fractional anisotropy (Eq. (G.1)) is a single estimation of the anisotropy degree using average ($c_{L_{mean}}$), maximum ($c_{L_{max}}$), and minimum ($c_{L_{min}}$) phase speed values at each location [4].

$$NFA_{location} = \sqrt{\frac{(c_{L_{max}} - c_{L_{mean}})^2 + (c_{L_{min}} - c_{L_{mean}})^2}{{c_{L_{max}}}^2 - {c_{L_{min}}}^2}} \quad \text{(G.1)}$$

The modified planar anisotropy coefficient $MPAC_\theta$ (see Eq. (G.2) [4]) uses phase speed as a measure of the planar-transverse strain ratio, at each semi-axis θ_i:

$$MPAC_{\theta_i} = \frac{1}{2}(c_{L_{\theta_i}} - 2\, c_{L_{\theta_i + 45^\circ}} + c_{L_{\theta_i + 90^\circ}})$$

(G.2)

H. OCT images from OCE measurements

The figures show the corneal (Fig. 5) and scleral (Fig. 6) OCT images of the same eye during OCE measurements. The ACUS excitation point was located in the center of each image as shown in Figure 5b and 5b (see also Fig. 2a in main manuscript). Figure 7 shows images of the wave propagation (multicolored pattern) during a measurement along scleral tissue.

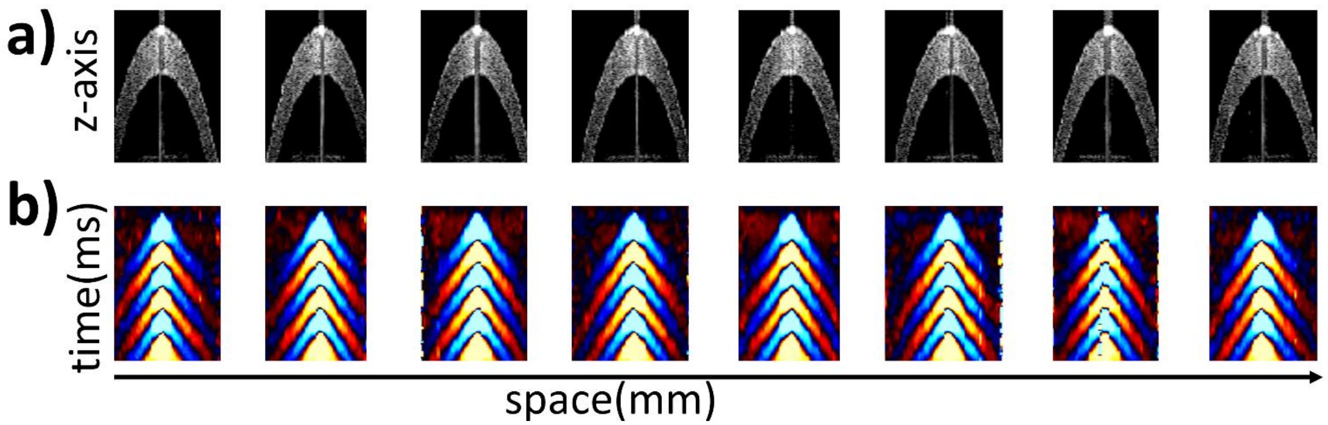

Fig. 5. ***Corneal images during OCE measurements.*** **a)** B-mode structural images of an *ex vivo* rabbit eye in the OCT field-of-view at central cornea (C) location. **b)** Corresponding space-time map in each of the 8 radial directions (16 semi-axes).

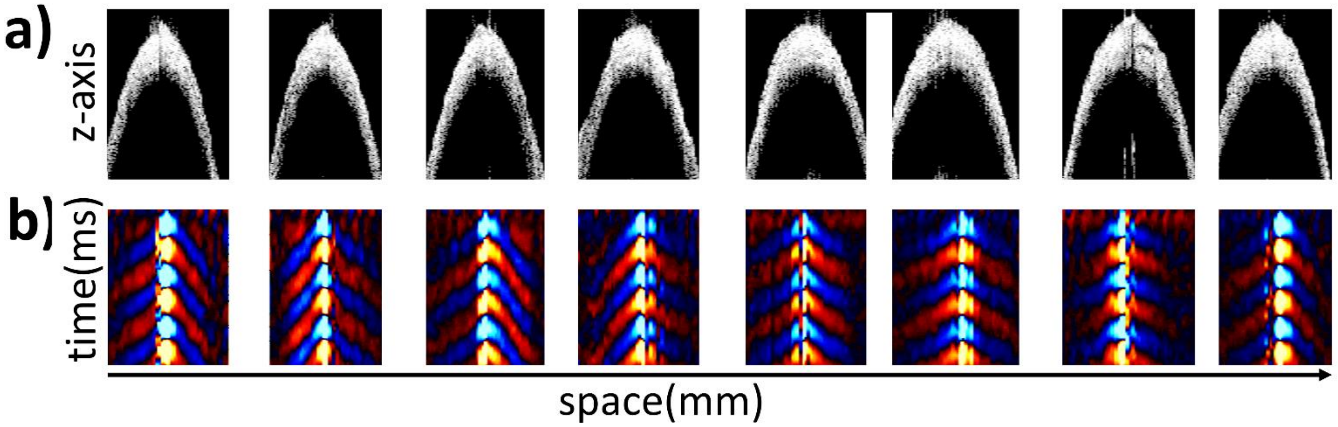

Fig. 6. ***Scleral images during OCE measurements.*** **a)** B-mode structural images of the rabbit sclera in the OCT field-of-view at the IN (Inferior Nasal) location, and **(b)** their corresponding space-time maps in each of the 8 radial directions (16 semi-axes).

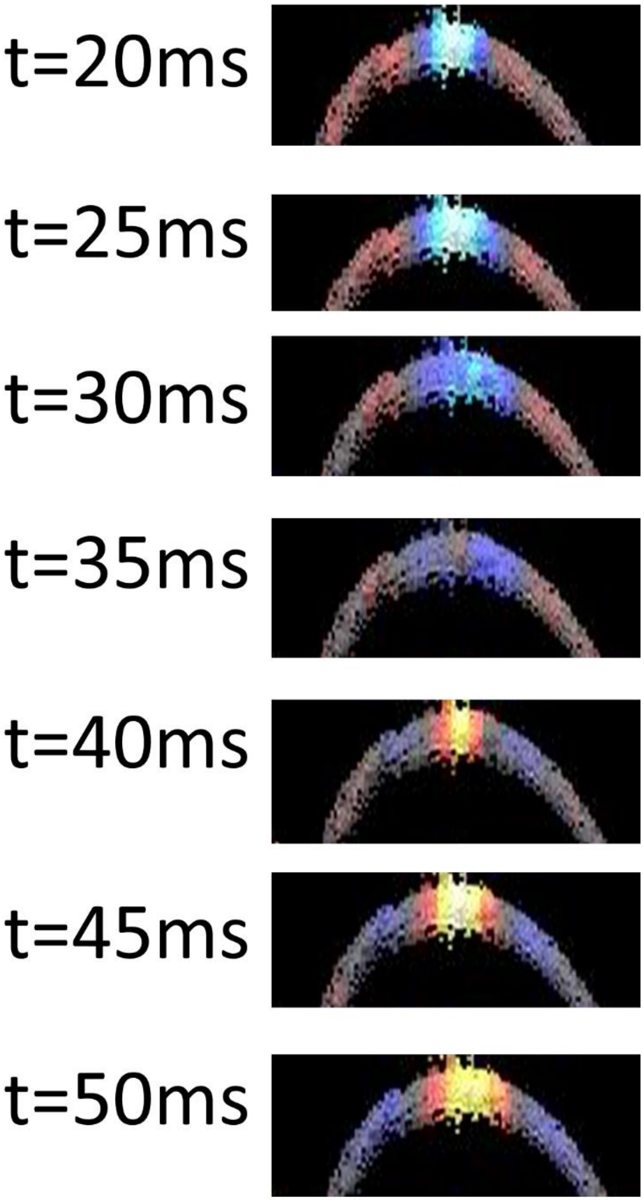

Fig. 7. ***Wave propagation in scleral OCT images of a rabbit eye.*** Wave propagation recorded over time in the OCE field-of-view in one meridian for IN (Inferior Nasal) scleral location of a rabbit eye at IOP of 15 mmHg.

Supplementary References

[1] A. Elsheikh, B. Geraghty, D. Alhasso, J. Knappett, M. Campanelli, P. Rama, Regional variation in the biomechanical properties of the human sclera, Exp. Eye Res. 90 (2010) 624–633. https://doi.org/10.1016/j.exer.2010.02.010.

[2] A. Barathi, M.K. Thu, R.W. Beuerman, Dimensional Growth of the Rabbit Eye, Cells Tissues Organs. 171 (2002) 276–285. https://doi.org/10.1159/000063123.

[3] L. Thomsen, Weak elastic anisotropy, Geophysics. 51 (1986) 1954–1966. https://doi.org/10.1190/1.1442051.

[4] M. Singh, J. Li, Z. Han, C. Wu, S.R. Aglyamov, M.D. Twa, K. V Larin, Investigating Elastic Anisotropy of the Porcine Cornea as a Function of Intraocular Pressure With Optical Coherence Elastography, J. Refract. Surg. 32 (2016) 562–567. https://doi.org/10.3928/1081597X-20160520-01.
